# Association between vitamin D serum levels and thyroid cancer: a meta-analysis

**DOI:** 10.3389/fendo.2025.1602844

**Published:** 2025-07-24

**Authors:** Lili Yang, Peng Yun, Fangping Li

**Affiliations:** Department of Endocrinology, The Seventh Affiliated Hospital, Sun Yat-Sen University, Shenzhen, China

**Keywords:** vitamin D level, vitamin d deficiency, thyroid cancer, meta-analysis, subgroup analyses

## Abstract

**Background:**

Thyroid cancer (TC) has shown a rising prevalence worldwide. While numerous studies have explored the relationship between vitamin D levels and TC risk, their conclusions remain inconsistent.

**Objective:**

This meta-analysis aims to evaluate the association between serum vitamin D levels, vitamin D deficiency, and TC based on existing evidence.

**Methods:**

We systematically searched the Embase, Web of Science, and PubMed databases for human studies investigating the relationship between vitamin D and TC including a control group. A random-effects model with forest plots was employed to calculate the mean difference (MD) in serum vitamin D levels, the odds ratio (OR) for vitamin D deficiency, and the risk difference (RD) between TC cases and controls. Meta-regressions and subgroup analyses were conducted based on the season of serum 25(OH)D sampling, source of controls, timing of measurement, study type, and testing methods of 25(OH)D. A *p*-value <0.05 was considered statistically significant.

**Results:**

A total of 23 studies were included. The meta-analysis revealed that TC patients had significantly lower serum vitamin D compared to the controls [SMD = −0.38 (95% CI: −0.62 to −0.14)].Additionally, vitamin D deficiency was significantly more prevalent among TC patients (OR = 1.33, 95% CI: 1.02 to 1.73, *P* < 0.05). The subgroup analyses demonstrated significant differences across most subgroups, except for post-operative measurements. Seasonal variation in 25(OH)D sampling was identified as a key source of heterogeneity.

**Conclusions:**

The meta-analysis suggests that lower serum vitamin D levels and vitamin D deficiency are significantly associated with an increased risk of TC. However, further studies with standardized protocols for seasonal sampling of vitamin D, source of control, measurement timing, study type, and testing methods of 25(OH)D are needed to clarify this relationship and its underlying mechanisms.

## 1 Introduction

Thyroid cancer is becoming increasingly prevalent worldwide, ranking as the seventh most common cancer among women in the United States and the ninth globally as of 2023 (1). While differentiated thyroid cancer (DTC) generally has a favorable prognosis and low mortality, medullary thyroid cancer (MTC) and especially anaplastic thyroid cancer (ATC) are associated with poor outcomes despite advancements in therapy (1).Hence, substantial challenges persist in the prevention, optimal management, and early detection of suboptimal outcomes. Vitamin D, a fat-soluble vitamin synthesized in the epidermis of the skin via the energy of ultraviolet radiation (UV-B) or obtained via diet or supplements, regulates more than 1,000 genes in a wide assembly of different cells and tissues involved in malignant cells’ biochemical pathways (2, 3). Vitamin D also regulates immune responses, cell proliferation, differentiation, and apoptosis (3). Therefore, the role of vitamin D in influencing malignant and tumor cells is undeniable. It is found to be a crucial factor in cancer pathology through its regulatory and metabolic roles in the body (4).

The pathogenesis and progression of thyroid cancer are still under discussion. Some scholars believed that potential risk factors for TC include chemical toxins, insulin resistance, metabolic syndrome, and vitamin D deficiency (5–7). There are a lot of studies about the risk of TC and vitamin D. Some studies found that vitamin D levels are significantly associated with the risk of TC and may be a protective factor in the development of TC (8). However, the conclusions are mixed (9–11). Moreover, seasonal variations in sunlight exposure, which affects vitamin D3 synthesis, may affect the results. At the same time, age, gender, ethnicity, types of thyroid cancer, timing of measurement, study type, and testing methods of vitamin D further complicate these associations. There was no unified attention on season and other factors mentioned above in previous meta-analysis. This meta-analysis aims to consolidate existing evidence and explore the impact of these variables on the vitamin D and TC relationship.

## 2 Methods

### 2.1 Literature search and extraction strategy

PubMed, Web of Science, and Embase were searched from their respective inception to September 28, 2024. The search terms used included (“thyroid cancer[Title/Abstract]” OR “thyroid tumor[Title/Abstract]” OR “thyroid carcinoma[Title/Abstract]” OR “TC[Title/Abstract]” OR “PTC[Title/Abstract]” OR “FTC[Title/Abstract]” OR “MTC[Title/Abstract]” OR “ATC[Title/Abstract]”) AND (“25-hydroxyvitamin D[Title/Abstract]” OR “vitamin D[Title/Abstract]” OR “25(OH)D[Title/Abstract]” OR “(25 OHD[Title/Abstract]” OR “cholecalciferol[Title/Abstract]”).

### 2.2 Eligibility criteria and study selection

The criteria for inclusion were as follows (1): randomized clinical trials (RCT), non-randomized clinical trial, observational studies (including cohort and case–control studies), or cross-sectional studies, (2) studies published in English, (3) studies conducted in human subjects, (4) patients with TC in the case group and healthy individuals (or those with benign thyroid diseases) in the control group, and (5) availability of complete data. We excluded the following types of studies: animal models, *in vitro* studies, narrative and systematic reviews, opinion papers, case reports, or abstract and conference papers.

### 2.3 Literature screening and data extraction

The following information was extracted independently using a predesigned form: study design, first author, year of publication, location, sample size, study population characteristics (number, age), vitamin D status, main statistical analysis methods, and, if applicable, adjustment variables included in statistical models, levels of vitamin D in both TC cases and controls, and adjusted odds ratios (OR) with 95% confidence intervals (CIs) for every category of vitamin D levels. Continuous variables are depicted as mean and standard deviation (SD); categorical variables are depicted as number and percentages.

### 2.4 Risk of Bias Analysis and Certainty of Evidence

The study quality was evaluated using the Newcastle–Ottawa Scale (NOS) for case–control studies, and the scale of the Agency for Healthcare Research and Quality (AHQR) was applied to cross-sectional studies.

### 2.5 Statistical analysis

Meta-analysis was performed using STATA software (version 15.0). According to whether the heterogeneity was low (*I* ^2^ < 50%) or high (*I* ^2^≥ 50%), we used fixed- or random-effects models, respectively. The OR was used as a summary statistic for dichotomous variables. The 95% CI was calculated for all mean values. Statistical significance was set at *p ≤*0.05, with *p*-values >0.05 considered non-significant.

## 3 Result

### 3.1 Literature search, study characteristics, and quality assessment

**Figure 1:**
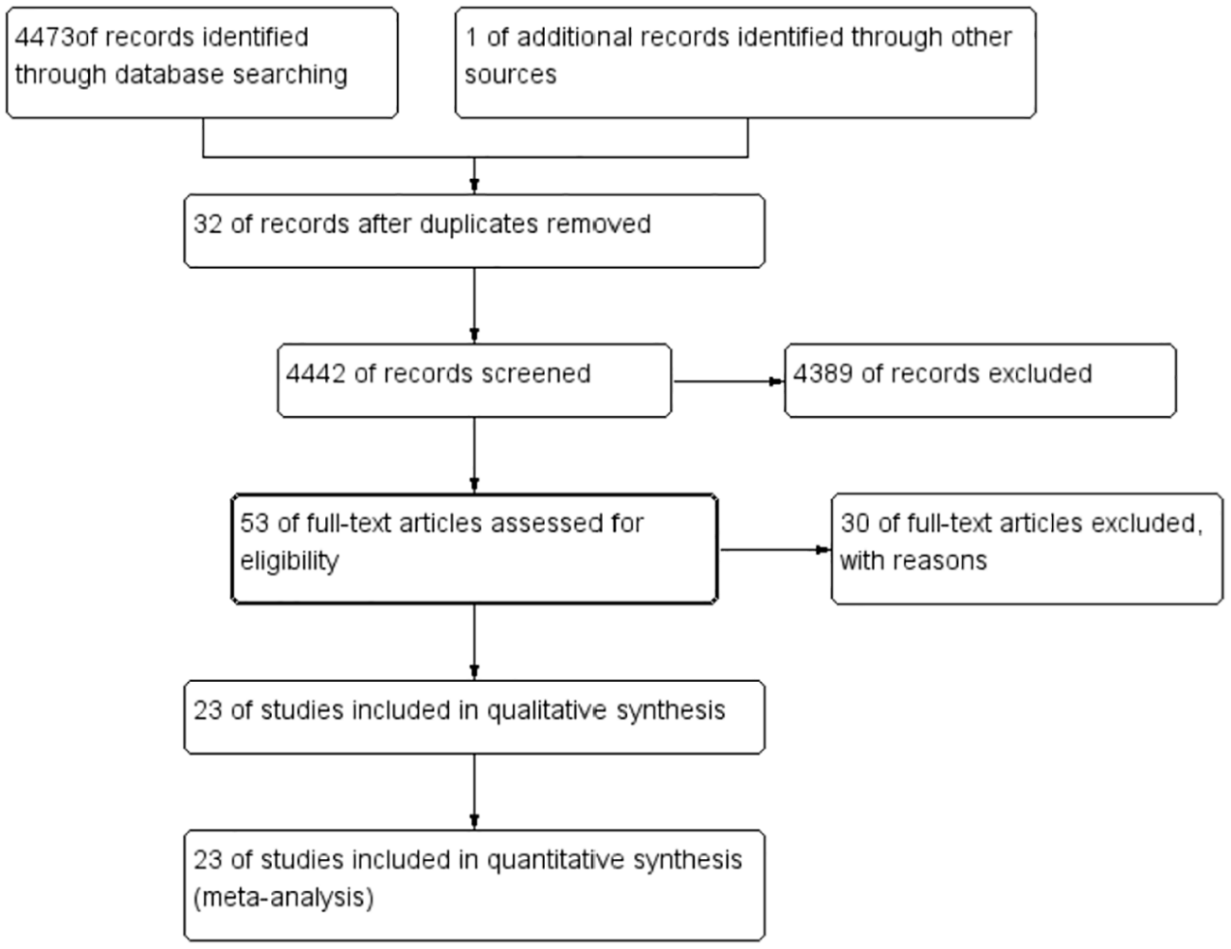
PRISMA flow diagram of the study.

A total of 4,474 were identified in the initial examination. After removing duplicates and conducting a systematic screening of titles, abstracts, and full texts, 23 studies meeting the inclusion criteria were selected for final analysis, comprising 18 case–control studies (12–29) and five cross-sectional studies (11, 30–33). The literature search process and results are shown in **Figure 1**. The 23 included articles comprised 12,153 participants (ranging from 30 to 5,186), 4,362 cases, and 7,791 controls. The included studies were published between 1982 (33) and 2024 (11) with geographical distribution as follows: China (*n* = 5) (12, 18, 20, 26, 28), Iran (*n* = 4) (14, 24, 27, 31), Romania (*n* = 2) (23, 30), and Turkey (*n* = 2) (19, 32) and one study each from the USA (13), Poland (15), Serbia (16), Korea (17), Germany (21), MS (22), Japan (33), Brazil (25), Greece (15), and Finland (30). Detailed characteristics of the 23 included studies are presented in **Table 1**. The quality of all included studies was relatively high, with quality scores ranging from 6 to 9 (**Tables 2**, **3**).

**Table 1:** Characteristics of the included studies.

First author	Year of publication	Country	Type of thyroid cancer	Source of controls	Case	Control	Age case	Age control	Female (%) case	Female (%) control	Seasons	Testing methods
Jie Kuang (12)	2022	China	PTC	Benign thyroid disease	1,578	128	43.97 ± 12.51	52.70 ± 13.48	77.2	78.9	May 2017 to June 2018	HPLC
A.M. Cocolos (30)	2022	Romania	PTC,FTC	Benign thyroid disease	170	200	49.90 ± 14.43	54.65 ± 11.67	81.76	84.5	Not mentioned	ECLIA
Zahra Heidari (14)	2017	Iran	DTC	Healthy euthyroid control and normal thyroid sonography participants	85	85	31.24± 11.36	31.94 ± 12.27	85	85	January 2013 to June 2016	EIA
Tomasz Stepien (15)	2010	Poland	PTC,FTC,ATC	Patients with multinodular nontoxic goiter; healthy volunteers	50	34; 26	54.15± 11.6	56.8 ± 14.3; 55.7± 9.2	66	67	Not mentioned	EIA
Ana (Vojislav) Koljevi (16)	2023	Serbia	DTC	Healthy individuals	105	34	61 (33–87)	44.5 (35–65)	90	94	January 2017 to December 2020	QEFA
Yun Mi Choi (17)	2017	Korea	PTC, FTC, MTC, and PTC or suspicious for malignancy by FNAC but did not undergo surgery	Healthy controls	53	5,133	54.4 ± 10.3	54.1 ± 9.1	36.5	37.7	Spring (March to May), summer (June to August), fall (September to November), and winter (December to February)	RIA
Ting Zhang (18)	2018	China	PTC	Benign thyroid disease	78	80	Not mentioned	Not mentioned	Not mentioned	Not mentioned	winter	ELISA
Ali Emami M. Sc (31).	2017	Iran	MTC	Healthy controls	40	40	40-49	40-49	37.5	37.5	Not mentioned	ELISA
Mustafa S¸ahin (19)	2013	Turkey	DTC	Not mentioned	344	166	45.5 ± 11	44.9 ± 8	85.3	84	between July 10, 2010 and January 1, 2012	ELISA
M.-J. Hu (20)	2020	China	PTC, FTC, MTC, ATC	Healthy control	506	506	49.4 ± 13.6	50.0 ± 13.4	65.61	65.61	May 2017 to March 2019	RIA
Marissa Penna-Martinez (21)	2012	Germany	PTC, FTC	Healthy controls without a family history of thyroid carcinoma	253	302	55	38	81.46	45.69	October 2006 to December 2008 and from April 2007 to September 2009, respectively	RIA
Jacqueline Jonklaas (22)	2013	Maryland	DTC	Benign thyroid disease	48	17	45.9 ± 13.5	52.5 ± 12.8	Not mentioned	Not mentioned	During the period 2008–2010	LC-MS
ANDRA-MARIA COCOLOS (23)	2022	Romania	PTC, FTC	Not mentioned	113	150	50 ± 14.46	55.87 ± 11.67	80.56	91.33	Between 2018 and 2020	CLIA
Mehmet Taner Unlu (32)	2023	Turkiye	PTC	Benign thyroid disease	119	103	46.8 ± 13.8	48.1 ± 12.5	77.31	82.52	Between 2012 and 2017	Not mentioned
Ali Kachui (24)	2017	Iran	PTC	Healthy relatives of patients who took no medications were included after BMI, sex, and age matching	34	38	F: 49.7 ± 38 M: 65.6 ± 7.33	F: 18.8 ± 2.36 M: 13.8 ± 2.33	74	74	April 2013 to March 2014	ELISA
Shoushi Lee (33)	1982	Japan	MTC	Health control	8	22	46 (14 68)	21 to 39	75	27.3	Not mentioned	CPBA
Debora Lucia Seguro Danilovic (25)	2016	Brazil	PTC,FTC	Benign thyroid disease	199	234	51.6 ± 15	54.6 ± 14.5	86	93	2009 to 2012	CLIA
Daqi Zhang (26)	2023	China	PTC	Benign thyroid disease	51	49	41 [35–50]	49 [44–55]	76.47	61.22	September 2018 and October 2019	LC-MS/MS
Nathan Laney (13)	2010	USA	PTC, FTC, follicular variant of papillary, or Hürthle cell thyroid	Patients with thyroid nodules	69	42	Not mentioned	Not mentioned	81.16	90	Not mentioned	CLIA OR: LC-MS/MS
S Lanitis (11)	2024	Greece	DTC	Benign thyroid disease	183	144	53.09 ± 13.68	54.64 ± 15.09	Not mentioned	Not mentioned	January 2017 and January 2020	Not mentioned
M Ramezani (27)	2020	Iran	MTC	Healthy control	40	40	36 ± 7.57	33 ± 6.02	65	55	Not mentioned	ELISA
M.-J. Hu (28)	2018	China	PTC	Healthy control	138	138	44.7 ± 13.4	44.0 ± 13.5			May 2017 to October 2017	RIA
Juka Toivonen (29)	1998	Finland	DTC	Healthy control	29	38	45 (27–71)	43 (26–65)	86.2	89.4	Not mentioned	SA-Kits

**Table 2:** Quality assessment for the included case–control studies according to the Newcastle–Ottawa Scale.

First author	Year of publication	Selection	Comparability	Exposure	Quality score
Jie Kuang (12)	2022	3	2	3	8
Nathan Laney (13)	2010	3	2	3	8
Zahra Heidari (14)	2017	3	2	3	8
Tomasz Stepien (15)	2010	3	2	3	8
Ana (Vojislav) Koljevi (16)	2023	3	0	3	6
Yun Mi Choi (17)	2017	4	1	3	8
Ting Zhang (18)	2018	4	0	2	6
Mustafa S¸ahin (19)	2013	3	2	3	8
M.-J. Hu (20)	2020	3	2	3	8
Marissa Penna-Martinez (21)	2012	3	0	3	6
Jacqueline Jonklaas (22)	2013	2	1	3	6
Andra-Maria Cocolos (23)	2022	3	1	3	7
Ali Kachui (24)	2017	3	2	3	8
Debora Lucia Seguro Danilovic (25)	2016	4	2	3	9
Daqi Zhang (26)	2023	3	0	3	6
M Ramezani (27)	2020	2	2	3	7
M.-J. Hu (28)	2018	2	2	3	7
Juka Toivonen (29)	1998	1	2	3	6

**Table 3:** Quality assessment for the included cross-sectional studies.

First author	A.M. Cocolos (30)	Ali Emami M. Sc (31).	Mehmet Taner Unlu (32)	Shoushi Lee (33)	S Lanitis (11)
Year	2022	2017	2023	1982	2024
Identify the source of information	Yes	Yes	Yes	Yes	Yes
List inclusion and exclusion criteria	Yes	Yes	Yes	Yes	Yes
Give the time period	Yes	Yes	Yes	Unclear	Yes
Whether the study subjects were continuous	Yes	Unclear	Yes	Unclear	Yes
Whether subjective factors mask other aspects of the study subject	No	No	No	No	No
Describe any evaluation conducted to ensure quality	Yes	Yes	Yes	Yes	Yes
Explain the rationale for excluding any patient from the analysis	Unclear	Yes	Unclear	Unclear	Unclear
Describe measures to evaluate and/or control confounders	Unclear	Yes	Unclear	No	Yes
Explain how lost data is handled in the analysis	Unclear	Unclear	Unclear	Unclear	Unclear
Summarize the response rate of patients and the completeness of data collection	Yes	Unclear	Yes	Unclear	Unclear
If there is follow-up, identify the percentage of patients with incomplete data or follow-up results.	Unclear	Unclear	Unclear	Unclear	Unclear

At the same time, we rigorously screened for duplicate datasets by comparing the authors, institutions, and participants’ demographics. Two studies from China (20, 28) shared overlapping authorship, but upon performing a meta-analysis, there are no repeated data. No other population overlaps were identified. Geographical clustering (e.g., five studies from China) reflects regional research activity rather than duplicate sampling.

### 3.2 Meta-analysis results

#### 3.2.1 Comparison of 25(OH)D3 levels in TC patients and controls

Among the 23 included studies, 19 reported serum 25-hydroxyvitamin D(25-OHD) levels in both TC patients and controls (11, 12, 14–20, 22–25, 27, 29–33). Due to variations in measurement units across studies, standardized mean difference (SMD) was used. In general, of the 19 studies, the serum vitamin D levels were significantly lower in TC patients.

**Figure 2:**
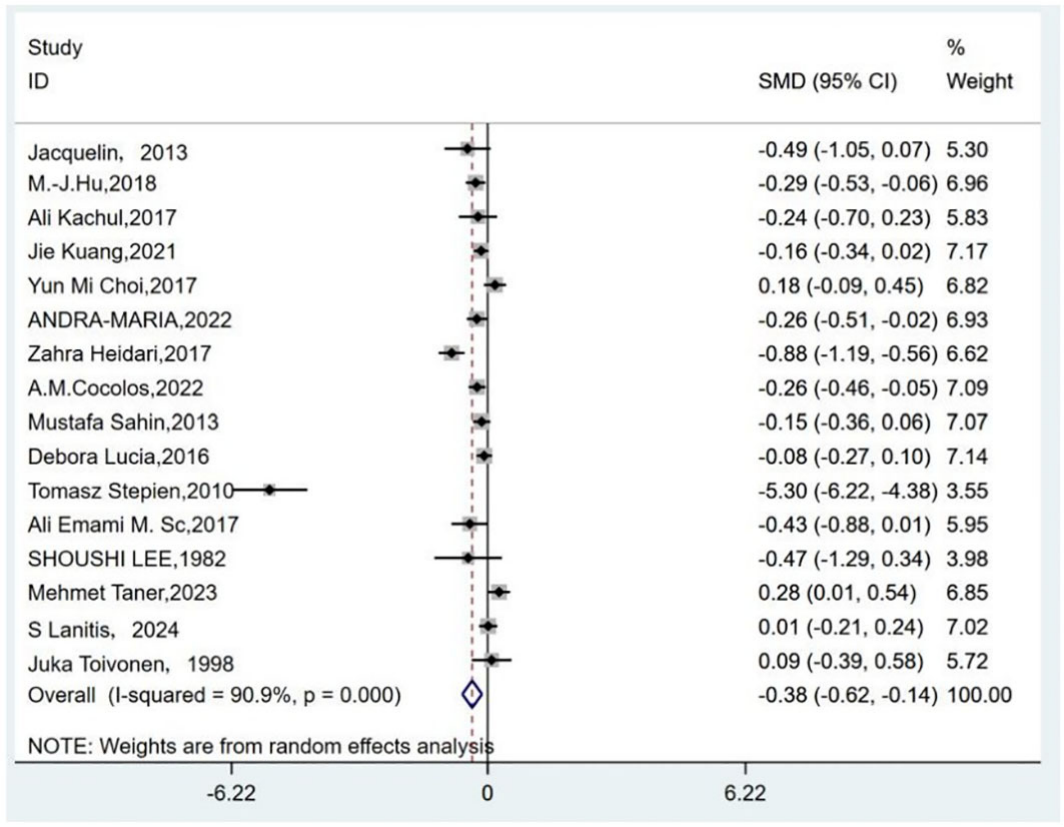
Forest plots of the standardized mean difference between the vitamin D levels in patients with thyroid cancer and control.

Owing to high heterogeneity (*I* ^2^ = 90.9%, *P* = 0.000), a random-effects model was adopted. A meta-analysis demonstrated a weighted mean difference of −0.38 (95% CI(−0.62 to−0.14), indicating significantly reduced 25(OH)D levels in TC patients (**Figure 2**). To investigate heterogeneity sources, a meta-regression analysis was performed, evaluating covariates including control group source, season of serum 25(OH)D sampling, thyroid cancer subtypes, timing of 25(OH)D measurement relative to diagnosis/treatment, and study design. Seasonal variation in blood sampling emerged as a significant contributor to heterogeneity (*P* = 0.046), while other factors showed no statistically significant associations (**Table 4**).

**Table 4:** Results of the meta-regression for the level of 25(OH)D.

Covariate	Coefficient	95% confidence interval	*P*-value
Source of controls	0.044	(-1.170, 1.259)	0.936
Seasons of obtaining a serum sample for 25(OH)D	-1.079	(-2.132,-0.026)	0.046
Types of TC	-0.149	(-0.846,0.548)	0.640
Timing of measurement	0.221	(-0.667,1.109)	0.587
Study type	1.624	-0.127,3.375)	0.065

The results of subgroup analyses stratified by control source, sampling season, cancer subtype, measurement timing, study type, and testing methods were not statistically significant in certain subgroups (**Table 5**).

**Table 5:** Results of the subgroup analysis for the SMD of 25(OH)D levels.

Characteristic	Subgroup	Number of studies	SMD (95% CI)	*I* ^2^	*P* for heterogeneity
Season of serum 25(OH)D sampling	All year round	10	-0.15 (-0.33, 0.02)	77.7%	0.000
	Summer and autumn	1	-0.29 (-0.53, -0.06)	–	–
	Not mentioned	5	-1.20 (-2.32, -0.08)	96.5%	0.000
Source of controls	Benign thyroid disease	6	-0.08 (-0.24, 0.07)	62.4%	0.021
	Healthy individuals	8	-0.82 (-1.44, -0.19)	95.0%	0.000
	Not mentioned	2	-0.20 (-0.36, -0.04)	0.00%	0.477
Timing of measurement	Pre-operation	8	-0.49 (-0.90, -0.09)	95.0%	0.000
	Post-operation	3	-0.22 (-0.42, -0.03)	0.0%	0.370
	Not mentioned	5	-0.42 (-0.71, -0.13)	73.3%	0.005
Study type	Case–control studies	11	-0.52 (-0.85, -0.19)	93.2%	0.000
	Cross-sectional studies	5	-0.11 (-0.37, 0.15)	71.2%	0.008
Testing methods	LC-MS	1	-0.49 (-1.05, 0.07)	–	–
	RIA	2	-0.06 (-0.52, 0.40)	84.8%	0.010
	HPLC	1	-0.16 (-0.34, 0.02)	–	–
	ECLIA	1	-0.26 (-0.46, -0.05)	–	–
	ELISA	3	-0.20 (-0.38, -0.03)	0.0%	0.510
	CLIA	2	-0.16 (-0.33, 0.02)	22.3%	0.257
	EIA	2	-3.06 (-7.39, 1.27)	98.7%	0.000
	CPBA	1	-0.47 (-1.29, 0.34)	–	–
	SA-Kits	1	0.09 (-0.39, 0.58)	–	–
	Not mentioned	2	0.13 (-0.12, 0.39)	55.5%	0.134

Subgroup analysis based on the pathological characteristics of thyroid cancer: A subgroup analysis was further conducted to evaluate the relationship between serum 25-hydroxyvitamin D (25[OH]D) levels and pathological characteristics (TNM stage and lymph node metastasis) in thyroid cancer (TC) patients. Limited studies addressed this association. According to the data that we summarized (**Table 6**), Tomasz Stepien et al. (15) reported significantly lower serum 25[OH]D levels in patients with advanced TNM stages, whereas Jie Kuang et al. (12) observed an inverse correlation with higher serum 25-OHD levels associated with advanced TNM staging. The remaining two studies (13, 16) showed no consistent trends in 25[OH]D levels relative to TNM stage or lymph node metastasis.

**Table 6:** Comparison of 25-OHD between thyroid cancer patients with different TNM stages.

First author	Year of publication	I	II	III	IV	*P*-value
Ana (Vojislav) Koljevi (16)	2023	16^a^	15^a^	24^a^	17^a^	
Tomasz Stepien (15)	2010	24.12 ± 6.77	16.93 ± 4.55	12.44 ± 8.98	6.18 ± 2.22	*P* < 0.05
Nathan Laney (13)	2010	84 (30–218)^a^	95 (85-108)^a^	80 (47–88)^a^	95 (45–95)^a^	0.71
Jie Kuang (12)	2022	17.15 ± 6.54		19.14 ± 7.41		<0.01

a Data are median (range).

**Figure 3:**
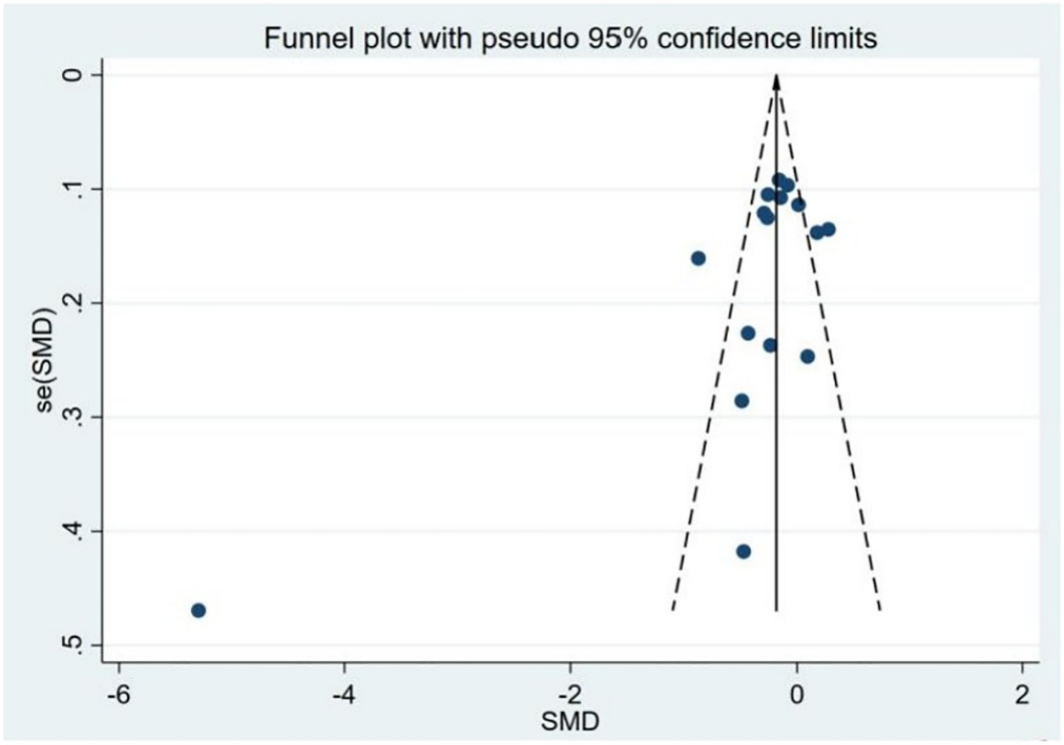
Funnel plot of the studies included in the meta-analysis of the standardized mean difference between the 25(OH)D levels in patients with thyroid cancer and controls.

**Figure 4:**
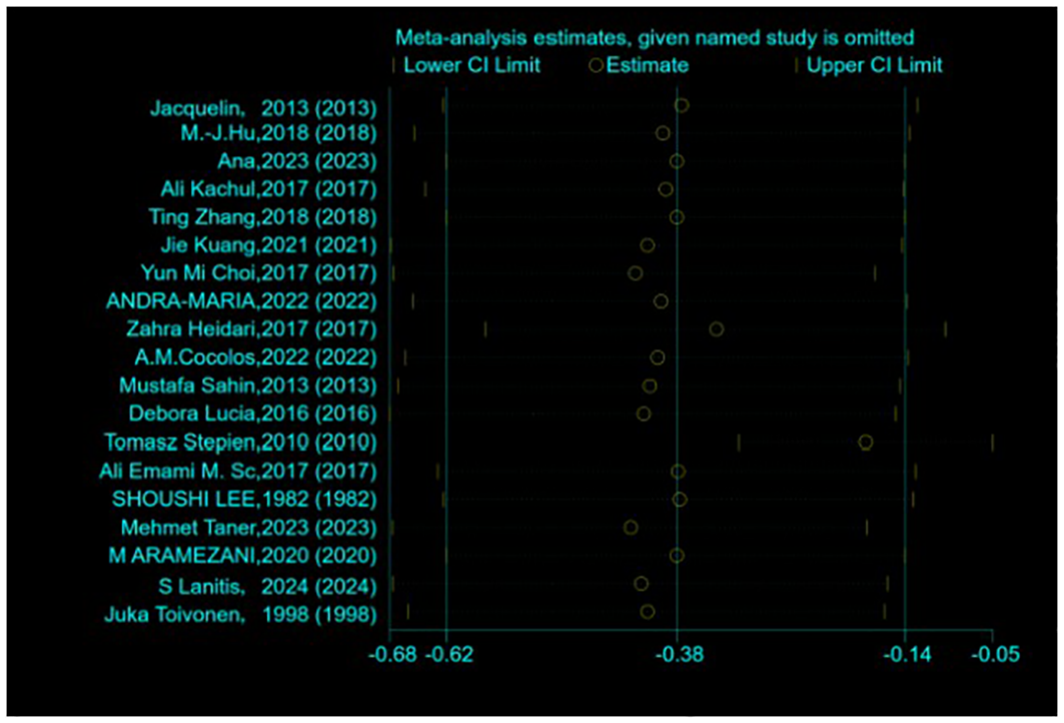
Sensitivity analysis of the standardized mean difference between the vitamin D levels in patients with thyroid cancer and control.

Assessment of publication bias and sensitivity analysis: Publication bias was assessed using funnel plots (**Figure 3**) and statistical tests. Begg’s tests did not reveal any significant publication bias (*P* = 0.192). However, Egger’s tests suggested a potential publication bias (*P* = 0.045). To address this discrepancy, a sensitivity analysis was conducted. The results of the sensitivity analysis showed that the removal of one trial (15) marginally influenced the effect size but did not alter the statistical significance or overall conclusions (**Figure 4**), confirming the robustness of our findings.

#### 3.2.2 Association between vitamin D deficiency and risk of TC

**Figure 5:**
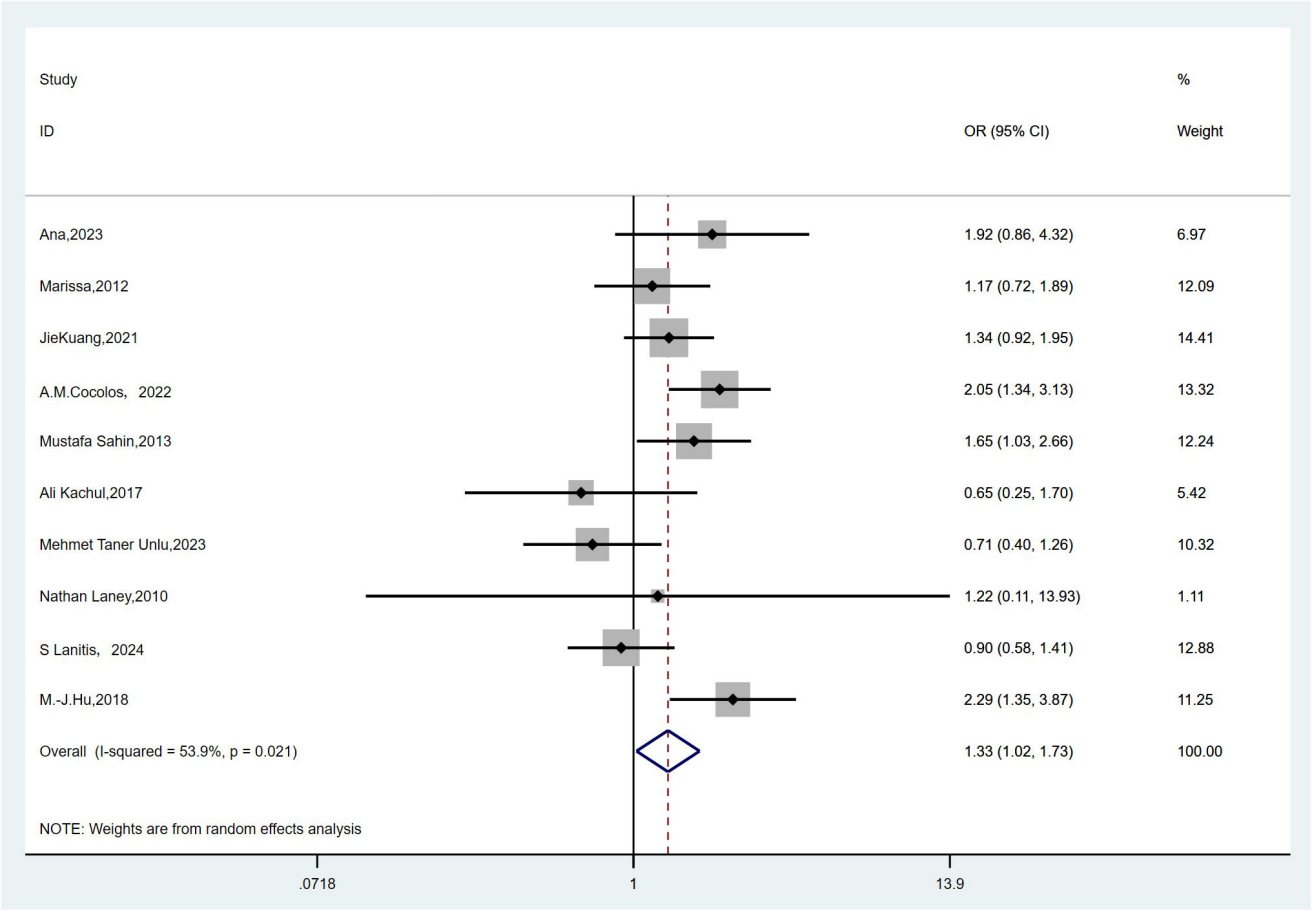
Forest plots and pooled estimates of the effect for the meta-analysis of the association between vitamin D deficiency and the risk of thyroid cancer.

A total of 10 studies reported the incidence of vitamin D deficiency in individuals with TC compared to the controls (11–13, 16, 19, 21, 24, 28, 30, 32). Owing to high heterogeneity (*I* ^2^ = 53.9%, *P* = 0.021), a random-effects model was adopted. The meta-analysis revealed that vitamin D deficiency was significantly associated with an increased risk of TC (pooled OR: 1.33, 95% CI(1.02–1.73), *P* < 0.05) (**Figure 5**). Vitamin D deficiency could increase the risk of thyroid cancer by 33% compared with individuals who are not deficient in vitamin D.

**Figure 6:**
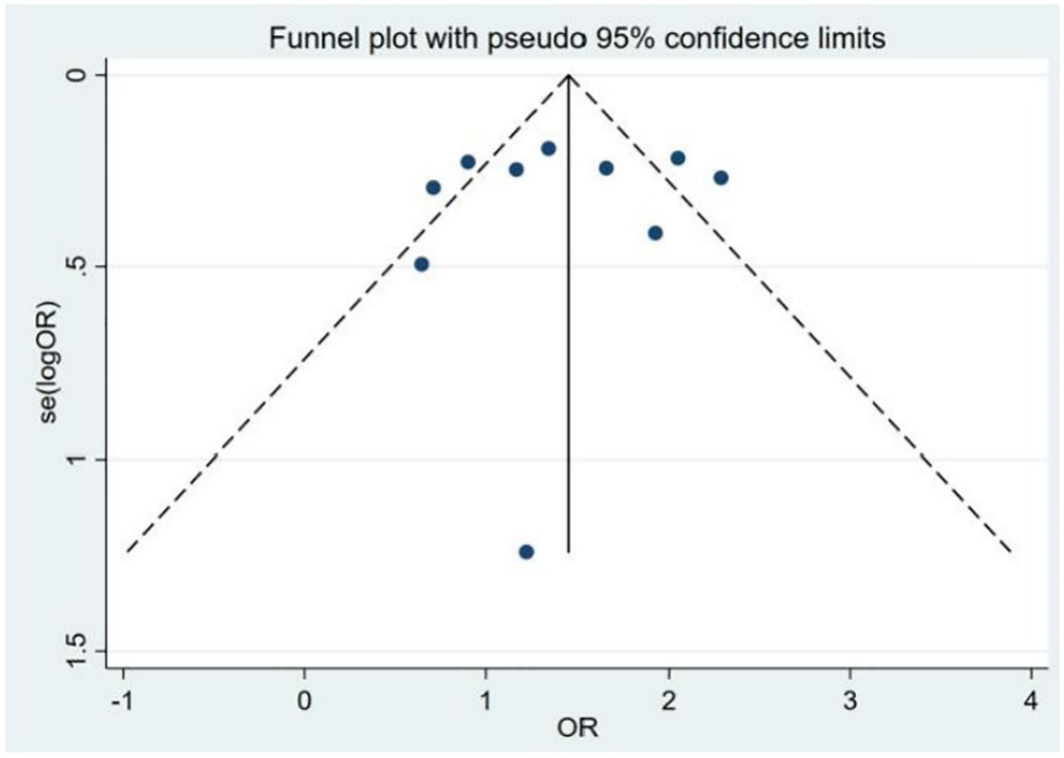
Funnel plot of the studies included in the meta-analysis of the deficiency of the 25(OH)D between patients with thyroid cancer and controls.

Because significant heterogeneity was detected, meta-regression was performed, evaluating potential influences from control source, season of serum 25(OH)D sampling, timing of measurement, and study type. As shown in **Table 7**, season of serum 25(OH)D sampling emerged as the only statistically significant moderator of heterogeneity (*P* = 0.020). Subsequent subgroup analyses further demonstrated that the association between vitamin D deficiency and TC risk was not statistically significant in certain subgroups (**Table 8**). Publication bias assessment through funnel plot visualization (**Figure 6**) combined with formal statistical testing (Egger’s test: *p* = 0.775; Begg’s tests: *p* = 0.858) indicated no substantial evidence of publication bias in the analyzed literature.

**Table 7:** Results of the meta-regression for the deficiency of 25(OH)D.

Covariate	Coefficient	95% confidence interval	P-value
Source of Controls	0.172	(-0.309,0.653)	0.376
seasons of obtain a serum sample for 25(OH)D	0.581	(0.151,1.011)	0.020
Types of TC	-0.791	(-2.378,0.796)	0.239
Timing of measurement	-0.168	(-0.615,0.280)	0.357
Study type	-0.476	-1.282,0.330	0.074

**Table 8:** Results of the subgroup analysis for the association between vitamin D deficiency and the risk of thyroid cancer.

Characteristic	Subgroup	Number of studies	OR (95% CI)	*I* ^2^	*P* for heterogeneity
Seasons of obtaining a serum sample for 25(OH)D	All year round	6	1.14 (0.83, 1.55)	48.2%	0.085
	Summer and autumn	1	2.29 (1.35, 3.87)	–	–
	Winter and spring	1	1.17 (0.72, 1.89)	–	–
	Not mentioned	2	2.02 (1.33, 3.07)	0.0%	0.682
Source of controls	Benign thyroid disease	5	1.19 (0.79, 1.78)	64.1%	0.025
	Healthy individuals	4	1.44 (0.88, 2.36)	55.7%	0.079
	Not mentioned	1	1.65 (1.03, 2.66)	–	–
Timing of measurement	Pre-operation	5	1.27 (0.89, 1.81)	67.4%	0.015
	Post-operation	4	1.56 (0.86, 2.84)	42.4%	0.157
	Not mentioned	1	1.17 (0.72, 1.89)	–	–
Study type	Case–control studies	7	1.47 (1.15, 1.88)	17.4%	0.297
	Cross-sectional studies	3	1.11 (0.59, 2.12)	81.6%	0.004
Testing methods	RIA	3	1.68(1.07, 2.63)	44.2%	0.167
	HPLC	1	1.34(0.92, 1.95)	–	–
	ECLIA	1	2.05 (1.34–3.13)	–	–
	ELISA	2	1.14 (0.46–2.81)	65.7%	0.088
	Not mentioned	2	0.82 (0.58–1.17)	0.00%	0.520
	CLIA	1	1.22 (0.11, 13.93)	–	–

## 4 Discussion

Vitamin D is well known for its association with calcium absorption and bone health. Pathologies due to vitamin D deficiency are characterized by hypocalcemia, hypophosphatemia, and dental and skeletal alterations. For several decades, it has been shown that vitamin D, in addition to maintaining bone and tooth health, also plays a key role in inflammatory and immune regulation (34). Vitamin D achieves this by interacting with immune cells and signaling pathways, ultimately contributing to the body’s ability to defend against infections and maintain immune homeostasis (35). Vitamin D and its metabolites are inexpensive natural compounds that may have a significant impact on the direct and indirect prevention of cancer (36). Increasingly, scholars are focusing on the relationship between vitamin D and tumors. Researchers believe that a low level of vitamin D is related to an increased risk of several cancers (37). Its anti-tumor effect is underpinned by a multitude of molecular mechanisms that impact cell growth, differentiation, and apoptosis (38). Given the increasing incidence of thyroid cancer, its pathogenesis and influencing factors have attracted increasing attention. The expression levels of the vitamin D receptor (VDR) and other genes involved in vitamin D signaling are increased in malignant thyroid cells (39, 40), suggesting a potential anti-tumor response of vitamin D in cancer. *In vivo* studies have shown that treatment with calcitriol reduces tumor size in mouse models of follicular thyroid cancer and metastatic follicular thyroid cancer (41). In human studies, some have shown that vitamin D deficiency may be a risk factor for thyroid cancer (14, 15, 30), while some studies have the opposite results or found no relevance (11, 32).

Our meta-analysis identified that TC was significantly associated with lower vitamin D, and the prevalence and odds of vitamin D deficiency are significantly higher among TC patients than among participants without TC. Eventually, the results of this study may be applied to clinical scenarios: attaining sufficient levels of vitamin D3 could be associated with a decreased risk of developing thyroid cancer.

However, our meta-analysis identified significant heterogeneity in the pooled estimates of serum 25(OH)D levels and vitamin D deficiency (*I*² = 90.9% and 53.9%, respectively). The results showed that seasonal variation in blood sampling was the source of the heterogeneity, indicating that it was necessary to obtain a serum sample for vitamin D in the same season to assess its impact on thyroid cancer. When it comes to subgroup analysis, there were limited studies that obtained a serum sample for 25(OH)D in the same season. M.−J. Hu et al. (28) reported that TC has a lower level of serum 25(OH)D when obtaining a serum sample in summer and autumn, which indicated that attention to and screening for vitamin D levels are also necessary during the summer and autumn seasons.

Although seasonal variation in blood sampling emerged as a key source of heterogeneity through subgroup analyses and meta-regression, further interpretation of its implications is warranted. The substantial heterogeneity may stem from methodological variations across studies (e.g., vitamin D measurement techniques, seasonal timing of sampling), population characteristics (e.g., geographic regions, BMI, sunlight exposure habit, dietary intake, history of vitamin D supplementation, and other factors), and diversity in study designs (case–control vs. cross-sectional studies). Despite the high heterogeneity, the subgroup analyses demonstrated consistent findings across all subgroups except for postoperative measurements, suggesting robustness in the primary conclusions.

Nevertheless, the observed heterogeneity may limit the generalizability of the results. Future research should prioritize standardized protocols for seasonal sampling, rigorous control of confounding variables, and harmonized methodologies to minimize heterogeneity and enhance comparability.

Our Egger’s test suggested a potential publication bias (*P* = 0.045), suggesting that smaller studies showing null or negative associations may remain unpublished. This bias could inflate the observed effect size. A sensitivity analysis excluding one outlier study marginally reduced the heterogeneity but did not alter the significance, supporting result robustness. Nonetheless, unpublished data gaps may limit the generalizability of our findings. We advocate for the prospective registration of observational studies to mitigate publication bias.

## 5 Limitations

There were some limitations in this meta-analysis. Firstly, we encountered substantial heterogeneity across the included studies. While we addressed this through detailed subgroup analyses based on various factors—with heterogeneity becoming statistically non-significant in some subgroups—the underlying variations remain a concern. Secondly, the cutoff values for 25-OHD varied considerably across eligible studies, complicating cross-study comparisons. Thirdly, Reza Tabrizi et al. (42) thought that, in addition to hypovitaminosis, VDR gene polymorphism can also negatively affect the action of vitamin D, and numerous studies have shown that the VDR level was increased in the case of DTC (mainly PTC was assessed) in comparison to the normal thyroid (18, 43). However, due to limited data, 25(OH)D and VDR polymorphisms were not involved in our meta-analysis. Meanwhile, given that all included studies in our meta-analysis were observational, causal relationships cannot be inferred. Consequently, more studies with standardized protocols for these factors are needed to better assess and clarify the mechanisms underlying our findings.

## Data Availability

The original contributions presented in the study are included in the article/supplementary material. Further inquiries can be directed to the corresponding author.
